# Multi-spectrum robotic cardiac surgery: Early outcomes

**DOI:** 10.1016/j.xjtc.2021.12.018

**Published:** 2022-02-19

**Authors:** Husam H. Balkhy, Sarah Nisivaco, Gianluca Torregrossa, Hiroto Kitahara, Brooke Patel, Kaitlin Grady, Charocka Coleman

**Affiliations:** Department of Cardiothoracic Surgery, University of Chicago Medicine, Chicago, Ill

**Keywords:** epicardial, intracardiac, minimally invasive, multi-spectrum, robotic cardiac surgery, TECAB, valve repair/replacement, EF, ejection fraction, ICU, intensive care unit, ITA, internal thoracic artery, LAA, left atrial appendage, LOS, length of stay, MV, mitral valve, PCI, percutaneous coronary intervention, STS, Society of Thoracic Surgeons, TECAB, totally endoscopic coronary artery bypass

## Abstract

**Objective:**

The robotic cardiac surgery program at our current institution began in 2013 with an experienced and dedicated team. This review analyzes early outcomes in the first 1103 patients.

**Methods:**

We reviewed all robotic procedures between July 2013 and February 2021. Primary outcomes were mortality and perioperative morbidity. Our robotic approach is totally endoscopic for all cases: off-pump for coronary and epicardial procedures, and on-pump with the endoballoon for mitral valve and other intracardiac procedures.

**Results:**

There were 1103 robotic-assisted cardiac surgeries over 7 years. A total of 585 (53%) were off-pump totally endoscopic coronary artery bypasses, 399 (36%) intracardiac cases (including isolated and concomitant mitral valve procedures, isolated tricuspid valve repair, CryoMaze, atrial or ventricular septal defect repair, benign cardiac tumor, septal myectomy, partial anomalous pulmonary venous drainage, and aortic valve replacement); 80 (7%) epicardial electrophysiology-related procedures (epicardial atrial fibrillation ablation, left atrial appendage ligation, lead placement, and ventricular tachycardia ablation); and 39 (4%) other epicardial procedures (pericardiectomy, unroofing myocardial bridge). Mortality was 1.2% (observed/expected ratio, 0.7). In the totally endoscopic coronary artery bypass and intracardiac groups, mortality was 1.0% (observed/expected, 0.6) and 1.5% (observed/expected, 0.87), respectively. There were 8 conversions to sternotomy (0.7%) and 24 (2.2%) take-backs for bleeding. Mean hospital and intensive care unit lengths of stay were 2.74 ± 1.26 days and 1.28 ± 0.57 days, respectively.

**Conclusions:**

This experience demonstrates that a robotic endoscopic approach can safely be used in a multitude of cardiac surgical procedures both on- and off-pump with excellent early outcomes. An experienced surgeon and team are necessary. Longer-term follow-up is warranted.

## Graphical abstract

Early outcomes in 1103 robotic-assisted endoscopic cardiac surgery cases at a single institution over 7 years.

**Figure:**
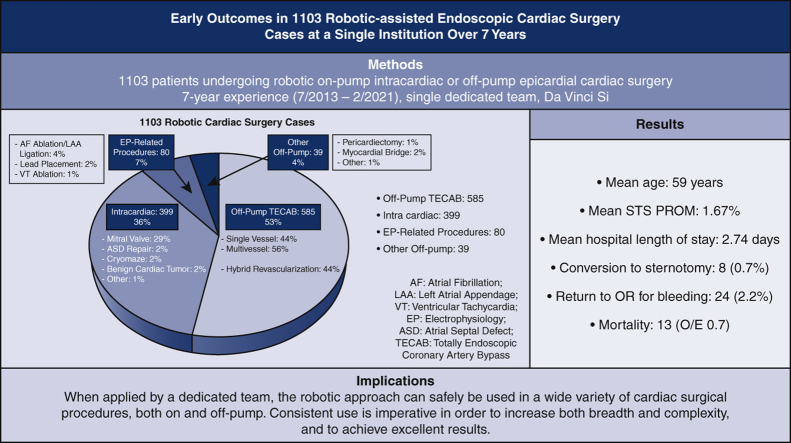

**Figure:**
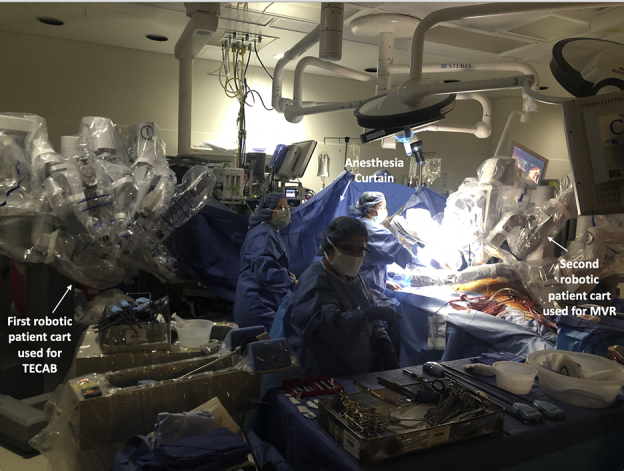
Operating room image: combined robotic TECAB and MV repair using 2 da Vinci (Intuitive) patient carts.

**Central Message**

When performed by a dedicated and experienced team, a wide range of cardiac surgical procedures can be conducted endoscopically using robotic assistance, with excellent outcomes.

**Perspective**

Robotic cardiac surgery for isolated coronary or MV disease is on the rise. Our program adopted robotics for multiple cardiac surgical indications as well as for patients with combined pathology. Our experience in 1103 patients over a 7-year period is presented and shows that with a dedicated team excellent early outcomes are possible.

The first clinical application of surgical robotics was in the late 1990s using an early prototype of the da Vinci robot (Intuitive), when the first mitral valve (MV) and coronary procedures were successfully completed in Europe.^1^, ^2^, ^3^ These were followed by Food and Drug Administration clearance in the United States, which paved the way for an increased use of the technology in mainly coronary and MV cases; however, the growth of the subspecialty remained fairly limited because of poor widespread adoption.^4^ The last 5 years have seen a significant rise in the adoption of robotic technology in cardiac surgery, especially in MV procedures, with the publication of several large series from high-volume centers showing excellent early outcomes.^5^^,^^6^ In coronary surgery, the use of the robot to harvest the internal thoracic artery (ITA) conduit has also increased among surgeons performing minimally invasive direct coronary artery bypass procedures.^7^^,^^8^

In the current era, most robotic cardiac programs continue to limit their procedures to coronary or MV repair cases. However, we believe that when a dedicated team uses a robotic approach on a routine basis, a paradigm shift can happen whereby a wide spectrum of various cardiac surgical procedures can be performed using a totally endoscopic approach.^9^ Additionally, inclusion/exclusion criteria can evolve with more experience to include increasingly complex and challenging cases, such as those patients who would benefit most from avoiding a sternotomy. In this study, we review the early outcomes of more than 1100 cases performed by a dedicated team at a single institution and describe our surgical approach to these procedures over a 7-year period.

## Patients and Methods

### Study Population

This is a single-center, retrospective study. Between July 2013 and February 2021, a total of 1103 consecutive patients underwent robotic cardiac surgeries by a single experienced surgeon and robotic team, including a dedicated bedside assistant throughout the experience (originally a registered nurse and later a physician assistant). We performed a retrospective review of our prospectively collected database with Institutional Review Board approval for this report (#18-0742; date of approval 4/28/2020). Individual patient consent was waived given this was a de-identified retrospective study. Preoperative characteristics, intraoperative data, and postoperative outcomes were analyzed. We used only eligible cases to calculate the Society of Thoracic Surgeons (STS) Predicted Risk of Mortality based on the STS calculator availability, which included totally endoscopic coronary artery bypass (TECAB), MV repair, aortic valve replacement, or a combination when appropriate. Only cases with associated STS scores were used to calculate the observed/expected mortality ratio. Primary outcomes were mortality and perioperative morbidity.

### Surgical Technique

All procedures were performed using the da Vinci Si Surgical robot.

### Epicardial Procedures

Almost all of our robotic epicardial procedures (including coronary bypass) are performed off-pump on the beating heart using a totally endoscopic approach with left-sided ports and the aid of the Intuitive Endowrist stabilizer. This instrument is placed through a subcostal port and is fully controlled by the console surgeon. We have found it indispensable as a coronary stabilizer in totally endoscopic coronary bypass (TECAB), especially when grafting the back of the heart, but we also use it as a positioner to help in conduit (single or bilateral ITA) harvesting in patients with limited intrathoracic space, as well as in exposing areas of the heart in cardiac ablation and left atrial appendage (LAA) ligation procedures.

### Intracardiac Procedures

When performing valve surgery (mitral, tricuspid, or aortic), biatrial Cryomaze procedures, septal defect repair, or benign tumor resection, right-sided ports are used with femoro-femoral cardiopulmonary bypass. We prefer the Intra-clude (Edwards Life Sciences) device for cardiac arrest in most cases, but use a Chitwood clamp with a percutaneous antegrade cardioplegia catheter in aortic valve cases or in patients not suitable for the Intra-clude device (eg, small peripheral vessels).

### Selection Criteria

Patients are referred directly to our robotic program or are self-referred seeking a sternal-sparing operation and therefore are self-selected for this approach. The only absolute exclusion criterion is fused pleural space secondary to prior thoracotomy or pulmonary resection on the relevant side. Relative contraindications include low ejection fraction (EF), significant pulmonary disease, and combined cardiac pathology. Otherwise, consideration for robotic surgery is on an all-comer basis. Our inclusion and exclusion criteria have evolved with increasing experience in the various procedures and with the increasing ability to offer a hybrid approach for patients with combined pathology. For example, redo cases are no longer an exclusion, and in patients with combined valve and coronary disease amenable to percutaneous coronary intervention (PCI), a hybrid strategy is offered. Inclusion criteria for TECAB are based on coronary anatomy in patients with one or two vessel disease, and candidacy for hybrid or advanced hybrid revascularization in patients with 3-vessel disease. Inclusion criteria for MV patients include all patients suitable for repair or replacement regardless of etiology.

### Statistical Analysis

Continuous variables are tested for normality. Those with normal distribution are expressed as mean ± standard deviation, and those without are expressed as median with interquartile range. Categorical and sequential variables are expressed as the number and percentage of patients.

## Results

A total of 1103 robotic-assisted cardiac surgeries were performed over 7 years.

### Patient Demographics

The mean age for all patients was 59 years (range, 18-91 years), 33% were female, and 6.5% were redo cardiac surgeries. Mean STS Predicted Risk of Mortality was 1.67 with a range of 0.2 to 28.0. Rates of hypertension, hyperlipidemia, and diabetes in the cohort were 73%, 61%, and 28%, respectively. A total of 18% had chronic kidney disease, and 3.4% were on dialysis. Rates of prior myocardial infarction, PCI, and stroke were 16%, 24%, and 8%, respectively. A total of 24% had atrial fibrillation preoperatively, 47% had a history of smoking, and 20% had congestive heart failure. A total of 16% had EF less than 40%, and mean EF was 51%. Full demographics are shown in Table 1.

**Table 1:** Patient demographics

Demographic variable	All patients (n = 1103)
Age (y), mean ± SD [range]	59 ± 14 [18-91]
Female gender, n (%)	362 (33)
BMI >30, n (%)	440 (40)
Hypertension, n (%)	801 (73)
Dyslipidemia, n (%)	671 (61)
Diabetes mellitus, n (%)	307 (28)
Peripheral vascular disease, n (%)	99 (8.0)
Chronic kidney disease, n (%)	202 (18)
Renal failure on dialysis, n (%)	38 (3.4)
COPD, n (%)	89 (8.1)
Smoking history, n (%)	514 (47)
Congestive heart failure, n (%)	223 (20)
EF (%), mean ± SD	51 ± 14
EF <40%, n (%)	172 (16)
Prior myocardial infarction, n (%)	181 (16)
Prior PCI, n (%)	270 (24)
Atrial fibrillation, n (%)	260 (24)
Prior CVA, n (%)	91 (8.2)
Previous cardiac surgery, n (%)	72 (6.5)
STS PROM score, mean ± SD (n = 902)	1.7 ± 2.4
STS PROM score range (n = 902)	0.15-28.0

*SD*, Standard deviation; *BMI*, body mass index; *COPD,* chronic obstructive pulmonary disease; *EF,* ejection fraction; *PCI,* percutaneous coronary intervention; *CVA,* cerebrovascular accident; *STS PROM,* Society of Thoracic Surgery Predicted Risk of Mortality.

### Case Breakdown

**Figure 1:**
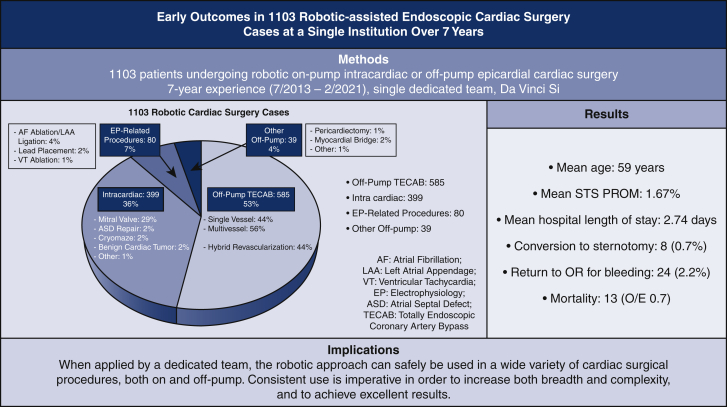
Summary of 1103 robotic endoscopic cardiac surgical procedures performed at a single institution over a 7-year period. The case breakdown is depicted on the left, and key outcomes are shown on the right. Implications of the study are summarized at the bottom. *STS PROM*, Society of Thoracic Surgery Predicted Risk of Mortality.

**Figure 2:**
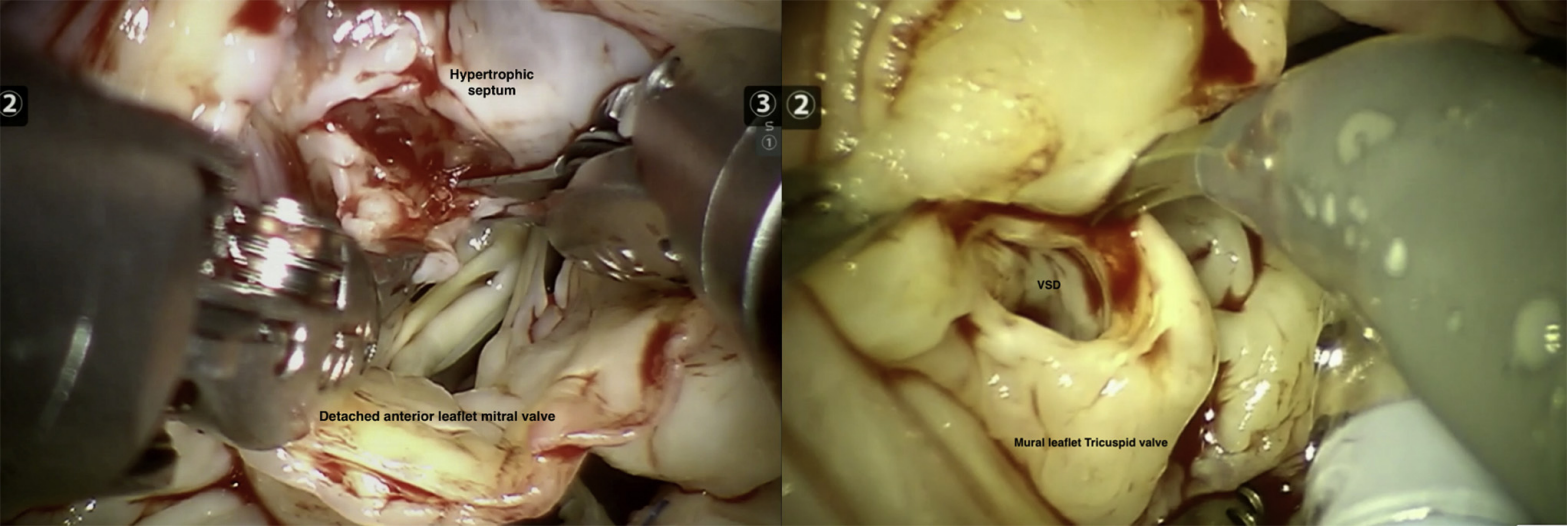
Two different examples of robotic-assisted intracardiac cases performed at our institution. Left: a robotic septal myomectomy for hypertrophic obstructive cardiomyopathy. Right: a robotic repair of a ventricular septal defect.

**Figure 3:**
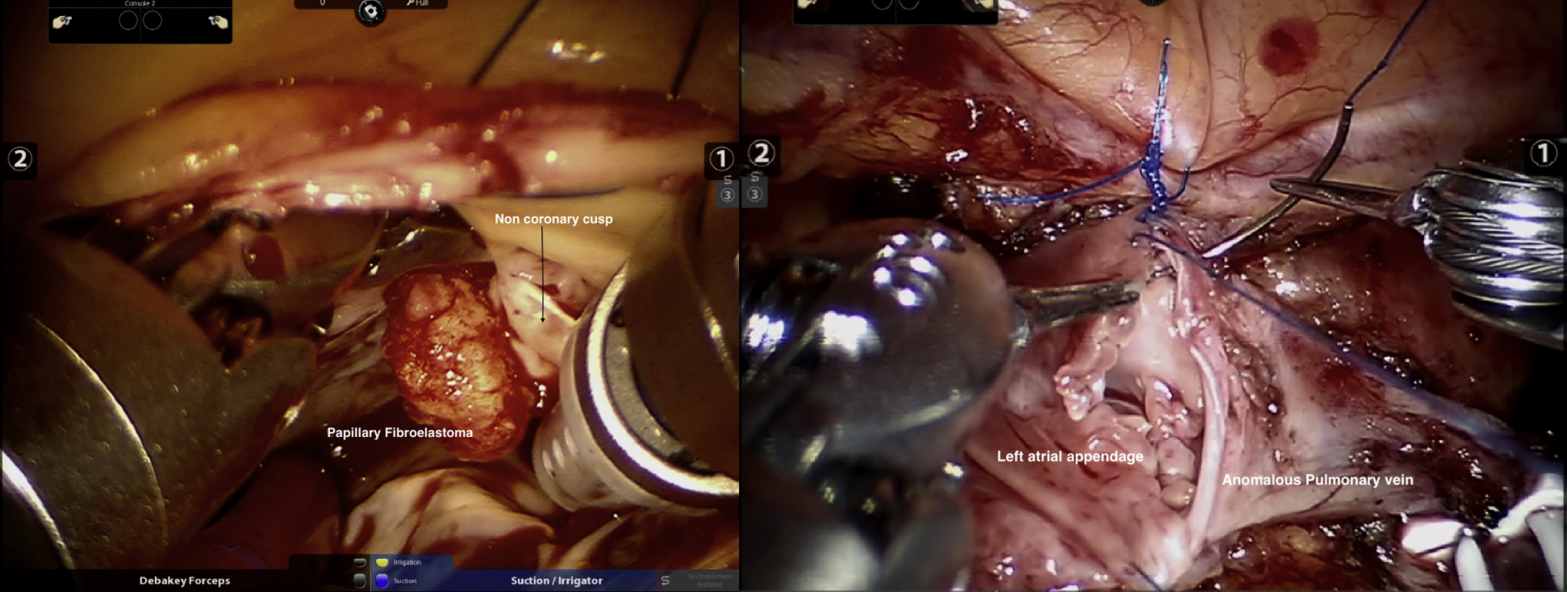
Two different examples of robotic-assisted intracardiac cases performed at our institution. Left: robotic resection of a papillary fibroelastoma found on the aortic valve. Right: a robotic anastomosis of the left anomalous pulmonary vein to the LAA during repair of left partial anomalous pulmonary venous return performed robotically.

The results of the whole cohort are described next and shown in Table 1, Table 2, Table 3, Table 4, Table 5. Results based on surgery type are shown in Table E1, Table E2, Table E3, Table E4. Case breakdown is shown in Figure 1. The largest subgroup in the cohort was composed of 585 (53%) off-pump TECABs (Video 1), followed by 399 (36%) intracardiac on-pump cases, 80 (7%) electrophysiology-related epicardial procedures, and 39 (4%) other off-pump epicardial cases. Examples of various robotic procedures are shown in Figures 2 and 3, including resection of an aortic valve mass, resection of hypertrophic obstructive cardiomyopathy (also demonstrated in Video 2), repair of a ventricular septal defect, and repair of left partial anomalous venous drainage, all done using a totally endoscopic approach.

**Table 2:** Intraoperative results

Intraoperative data	All patients (n = 1103)
Operative time (min), mean ± SD	185 ± 79
Concomitant procedure, n (%)	313 (28)
Intraoperative blood transfusion use, n (%)	143 (13)
Conversion to sternotomy, n (%)	8 (0.7)
Inotrope use, n (%)	44 (4.0)
Extubation in operating room, n (%)	368 (33)

*SD*, Standard deviation.

**Table 3:** Early postoperative outcomes

Postoperative variables	All patients (n = 1103)
Extubation <6 h, n (%)	864 (79)
Prolonged intubation (>24 h), n (%)	50 (4.5)
Reintubation, n (%)	26 (2.4)
24-h chest tube drainage (mL), mean ± SD	506 ± 362
Total chest tube drainage (mL), mean ± SD	818 ± 1052
Chest tube removal POD 1, n (%)	718 (65)
Postoperative blood transfusion use, n (%)	209 (19)
ICU LOS (d), mean ± SD	1.3 ± 0.6
Hospital LOS (d), mean ± SD	2.7 ± 1.3
Discharge destination	
Home, n (%)	1008 (91)
Rehabilitation facility, n (%)	82 (7)

*SD,* Standard deviation; *POD,* postoperative day; *ICU,* intensive care unit; *LOS,* length of stay.

**Table 4:** Early postoperative complications/30 day

Postoperative variables	All patients (n = 1103)
Atrial fibrillation, n (%)	134 (12)
Wound infection, n (%)	1 (0.09)
Groin complication, n (%)	8 (0.7)
Postoperative AKI, n (%)	27 (2.4)
Sepsis, n (%)	4 (0.4)
Pneumonia, n (%)	12 (1.1)
Pleural effusion, n (%)	41 (3.7)
Pneumothorax, n (%)	7 (0.6)
Pericarditis, n (%)	18 (1.6)
DVT, n (%)	8 (0.7)
PE, n (%)	4 (0.4)
Unilateral lung edema, n (%)	6 (0.5)
CHF, n (%)	8 (0.7)
Permanent pacemaker placement, n (%)	9 (0.8)
Tracheostomy, n (%)	11 (1.0)
ECMO, n (%)	17 (1.5)
Postoperative stroke, n (%)	7 (0.6)
Postoperative myocardial infarction, n (%)	2 (0.2)
Take-back for bleeding, n (%)	24 (2.2)
Postoperative sternotomy, n (%)	5 (0.5)
Mortality, n (%)	13 (1.2)
Mortality, O/E	0.70

*AKI*, Acute kidney injury; *DVT*, deep vein thrombosis; *PE*, pulmonary embolism; *CHF*, congestive heart failure; *ECMO,* extracorporeal membrane oxygenation; *O/E,* observed/expected; *SD,* standard deviation.

**Table 5:** Return to work/activities and postoperative opioid use

Postoperative variable	N = 285 patients
Last day of opioid medication after discharge (d), mean ± SD	4.9 ± 5.6
Patients taking no opioids postoperatively, n (%)	97 (34)
Patients off opioids within 1 wk, n (%)	227 (80)
Time to return to full normal activities (d), mean ± SD	14.9 ± 7.8
Return to full activities within 2 wk, n (%)	171 (60)
Time to return to work (d), mean ± SD	16.2 ± 12
Return to work within 2 wk, n (%)	61 (21)

*SD*, Standard deviation.

### Perioperative Outcomes

Mean operative time was 185 minutes. Eight patients (0.7%) required conversion to sternotomy. 13% required intraoperative blood transfusion, and 4% required inotropes. A total of 313 patients (28%) had concomitant procedures performed. Extubation in the operating room occurred in 33%. Intraoperative outcomes are shown in Table 2.

Postoperatively, 79% of patients were extubated within 6 hours. A total of 4.5% required prolonged intubation (>24 hours), and 2.4% required reintubation. A total of 65% had chest tube removal on postoperative day 1. Some 19% required blood transfusion postoperatively. Mean hospital and intensive care unit (ICU) length of stay (LOS) were 2.7 and 1.3 days, respectively. At discharge, 91% were discharged to home and 7% to acute or long-term rehabilitation facilities (Table 3).

Early postoperative complications are shown in Table 4. The incidence of new atrial fibrillation was 12%. There was 1 incidence of wound infection. 2.4% and 3.7% of patients had postoperative acute kidney injury and significant pleural effusion (requiring thoracentesis), respectively. Incidence of requirement for permanent pacemaker placement, tracheostomy, and extracorporeal membrane oxygenation were 0.8%, 1%, and 1.5%, respectively. There were 7 postoperative strokes (0.6%) and 2 clinical myocardial infarctions (0.2%). A total of 24 patients required take-back to the operating room for bleeding (2.2%); only 5 of these required sternotomy. Thirteen patients (1.2%) died, with an observed/expected ratio of 0.70.

Return to work/full activities and opioid use after surgery data were collected for the last 285 patients. Cessation of all opioid pain medications occurred at a mean of 5 days after discharge. A total of 34% took no postoperative opioid pain medication at home, and 80% had stopped taking any opioids within 1 week of discharge. Mean time to return to normal activities was 15 days, and return to work was 16 days. Data are listed in Table 5.

## Discussion

The benefits of performing coronary and MV procedures using a sternal-sparing robotic approach have been demonstrated and include less morbidity, early discharge after surgery, and quicker return to normal activities.^10^, ^11^, ^12^, ^13^ Our study demonstrates the feasibility of successfully using a robotic-assisted approach to facilitate endoscopic cardiac surgery for a multitude of indications. We have shown that when a dedicated surgeon and team use this technology on a routine basis, excellent early outcomes in a wide spectrum of both epicardial off-pump and intracardiac on-pump procedures can be achieved.

The application of robotic technology in surgery was a mere curiosity before the 1990s. With the introduction of the integrated Zeus robotic system and the da Vinci surgical system, clinical applications became possible in the latter half of the decade. Cardiac surgery was the intended target of both of these systems, and the first clinical procedures performed were on the heart.^3^^,^^14^ The 2 entities merged in the early 2000s. Around that time, it became clear that other (noncardiac) specialties would be quicker to adopt the technology. Over the ensuing decade and a half, the use of surgical robots became an integral part of urologic surgery. Slowly but surely, other specialties began to harness the technology for use in their procedures including gynecology, general surgery, thoracic surgery, and ear, nose and throat, among others.^15^^,^^16^ The few cardiac surgery programs, mainly in the United States, that continued to use a robotic approach did so in mitral^6^^,^^17^ or coronary^18^^,^^19^ surgery with excellent outcomes. Because of the pioneers in these programs, robotic cardiac surgery continued, although with little widespread adoption, especially outside the United States. The last 10 years have seen a renewed interest in this subspecialty,^4^ with several large programs in the United States adopting robotic MV repair^5^^,^^20^^,^^21^ and robotic minimally invasive direct coronary artery bypass^7^^,^^8^^,^^22^ successfully. In addition, multiple robotic programs have emerged in Europe over the last 5 years focusing on mitral or coronary applications.^23^

Our view has been that the efficiency and competence of the surgeon and team increases when the robot is used as frequently as possible. In our experience, we began using the robot in late 2007 and gradually increased our volume and variety of robotic cases after going through the initial learning curve to the extent that our team is now fully dedicated to this approach on a daily basis. The lead surgeon's learning curve in robotics occurred at a prior institution over more than 600 total cases (including ∼300 TECABs) over a 6-year period, before moving to the present institution. The experience described in this report began at the current institution in 2013, well past our learning curve. We were fortunate to have secured a dual console system for daily use with commitment from other important stakeholders, including hospital administration, nursing, and anesthesia. Having a dedicated team and robot allowed us to consider the technology as merely another operating room tool used routinely in a broad portfolio of procedures. We have previously published on the elements necessary to have a successful multifaceted robotic heart program including strong institutional support, a well-defined setup for each procedure, and development of collaborations with cardiology colleagues who not only understand the value of the robotic approach in the management of their patients but also can enhance the program by providing hybrid solutions.^9^

After starting with robotic ITA harvesting in patients with single-vessel coronary artery disease, we transitioned to single and multivessel TECAB while at the same time introducing intracardiac procedures such as atrial septal defects and simple mitral repairs. The latter was built on an extensive experience in sternal-sparing (nonrobotic) valve surgery such that the team was well past the learning curve of peripheral cardiopulmonary bypass and myocardial protection. We also worked to transition simple procedures we were performing thoracoscopically (eg, pericardial windows, epicardial lead placements, thoracoscopic LAA ligations) to a robotic approach to increase exposure and comfort with the robotic system for all involved. Subsequently, as the robot became an integral part of our workflow, we gradually started using it in more complex procedures, the basic surgical tenets of which were already mastered in the open setting. This included, for example, multi-arterial off-pump coronary bypass via sternotomy before multivessel TECAB and open complex mitral repairs before robotic complex MV repairs.

Most robotic programs focus on MV repair or ITA takedown with the robot. Our philosophy has always been that robotic technology is merely a tool that has the ability to make minimally invasive surgery easier. We believe that is true for all surgical specialties, including cardiac surgery. The aim of this study was to add detail to this overarching concept in describing the different procedures in a broad sense. A detailed discussion of each of the procedures performed is beyond the scope of this article. We have previously published on the detailed setup for all of the robotic procedures we perform.^9^

Demonstrating the routine use of robotics in the cardiac operating room made it justifiable for the hospital administration to allocate a dual console robot to the cardiac team on a daily basis. This allowed us to increase our volume and offer this approach to more patients with significantly lower hospital LOS and increasingly shorter wait times. In a recent study, Abbas and colleagues^24^ looked at the financial impact of integrating robotics at an academic program and demonstrated that high-acuity services such as thoracic surgery drive higher contribution margins as long as variable costs (especially hospital LOS) are kept low. We have looked at our internal cost data specifically for robotic TECAB at our institution and found that despite the higher intraoperative cost, the overall cost is favorable for TECAB because of the shorter ICU and hospital lengths of stay.

Yanagawa and colleagues^13^ reported on the benefits of a robotic approach in cardiac surgical procedures in a propensity-matched study published in 2015 looking at early outcomes and cost in more than 5000 patients undergoing a variety of robotic cardiac (mainly coronary and MV repair) procedures. They found that although the robotic approach was more costly, it was associated with significantly lower mortality, fewer complications, and shorter LOS when compared with a nonrobotic (sternotomy) approach.^13^ The mean LOS was 5 days, compared with 6 days in sternotomy, and using the robotic approach added on average $1531 to the procedure cost. The authors acknowledged that with more experience and efficiency using the robotic approach, hospital (and particularly ICU) LOS can further be reduced as has been shown in the orology literature with increased reduction in cost potentially offsetting this difference.^25^

The ICU and hospital LOS in our cohort were lower than in most reports of minimally invasive and robotic cardiac procedures.^26^, ^27^, ^28^ This is related not only to the less invasive and endoscopic nature of the surgery but also to the early recovery mindset of the postoperative care team. Although extubation in the operating room occurred in only 33% of patients, the mean LOS was less than 3 days. We have not yet performed a detailed cost analysis for this cohort of patients, but an important consideration (in addition to the fixed and variable intraoperative costs of the procedure in a busy hospital) is the opportunity cost of early discharge and thus increasing the capacity to treat more patients. The discharge destination in this cohort of patients is also notable where 91% of patients were discharged to home. When coupled with the shorter hospital stays associated with robotic surgery, this finding adds strength to the notion that the use of this technology is justified to reduce overall costs despite the noted increase in intraoperative costs.

Our study shows that return to work and full activity are significantly enhanced by the robotic approach (average time of 2 weeks). West and colleagues^29^ showed that patients undergoing minimally invasive coronary artery bypass grafting were more likely to return to employment compared with patients undergoing sternotomy coronary artery bypass grafting, and did so on average 6.6 weeks earlier than the sternotomy patients. Sternotomy has been shown to be associated with significantly higher rates of longer-term opioid use.^30^ In our cohort, use of opioids after surgery was reduced. More than 30% of our patients never filled their discharge prescription, and 80% were not using opioids after 1 week. We think these are some of the less appreciated aspects of the robotic approach that bear further study.

Our team is currently dedicated to applying a robotic approach for all appropriate indications, regardless of the patients’ perceived surgical risk, if we think they would benefit from a sternal-sparing approach. The definition of this changes with added experience and efficiency. As is well known, many of the patients who benefit most from a robotic approach are indeed the higher-risk patients (eg, frail, obese, redo). In collaboration with cardiology colleagues, we have also been able to customize the most appropriate intervention for each patient even in those with combined pathology. For example, decoupling coronary and valvular pathology in frail patients who may not tolerate sternotomy has led us to perform hybrid PCI and robotic mitral repair, or hybrid TECAB and TAVR in certain situations.

With added experience in the various traditional isolated procedures, we have shown that it is possible to combine robotic procedures, for example, TECAB and mitral repair,^31^ in highly selected patients. A total of 28% of patients in this cohort underwent concomitant procedures. The majority were the addition of a CryoMaze or tricuspid valve repair to a mitral case or the addition of LAA ligation to a TECAB. More recently, some have involved more complex combinations such as TECAB with MV repair or combined aortic and MV procedures. These combinations would have necessitated a sternotomy earlier in our experience. It is important to emphasize the gradual nature of evolution in these combined interventions that they entail extensive discussion with the patient within a multidisciplinary heart-team setting.

### Study Limitations

This is a retrospective single-center review study with all of the limitations inherent in a retrospective study design. The patients were selected by virtue of our institution being a robotic referral center, and there was no matched control group. The surgical team performing these procedures was experienced and well past the learning curve. Thus, these results cannot be expected to be reproduced in a less-experienced setting. Another limitation is that we reported only the early outcomes in these patients, and further longer-term follow-up will be necessary to validate these results.

## Conclusions

Our study demonstrates that a robotic totally endoscopic approach can safely and effectively be applied to a wide variety of cardiac surgical procedures, both on- and off-pump. In our view, an experienced, dedicated surgeon and team are necessary for both the breadth of cases and to achieve excellent results. Longer-term follow-up is warranted.

### Webcast

You can watch a Webcast of this AATS meeting presentation by going to: https://aats.blob.core.windows.net/media/21%20AM/AM21_A37/AM21_A37_03.mp4.

**Figure:**
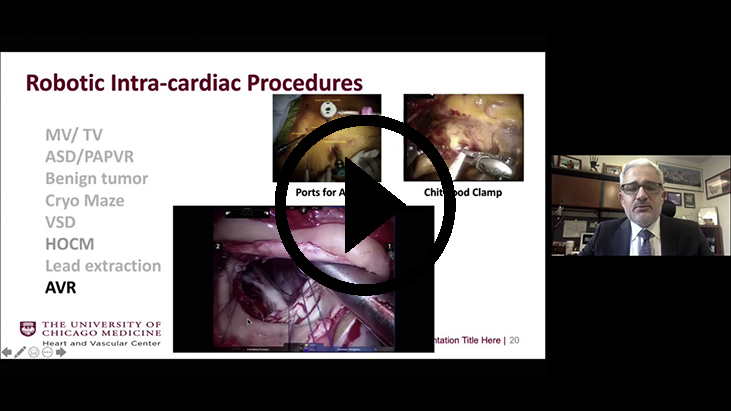

### Conflict of Interest Statement

H.H.B. discloses he is a proctor for Intuitive Surgical, maker of the da Vinci robot. All other authors reported no conflicts of interest.

The *Journal* policy requires editors and reviewers to disclose conflicts of interest and to decline handling or reviewing manuscripts for which they may have a conflict of interest. The editors and reviewers of this article have no conflicts of interest.

## Figures and Tables

**Table E1:** A total of 585 totally endoscopic coronary artery bypass cases (totally endoscopic coronary artery bypass)

Demographic variable	N = 585
Age, mean ± SD	65.7 ± 10.5
STS score, mean ± SD	1.65 ± 2.52
Intraoperative blood transfusion, n (%)	43 (7.35)
Extubation <6 h, n (%)	481 (82.2)
Hospital LOS (d), mean ± SD	2.69 ± 1.28
ICU LOS (d), mean ± SD	1.29 ± 0.68
Take-back for bleeding, n (%)	6 (1.03)
Conversion, n (%)	1 (0.18)
Postoperative stroke, n (%)	1 (0.18)
Postoperative MI, n (%)	1 (0.18)
Mortality, n (%)	6 (1.03)
Mortality, O/E	0.62

*SD,* Standard deviation; *STS,* Society of Thoracic Surgeons; *LOS,* length of stay; *ICU,* intensive care unit; *MI,* myocardial infarction; *O/E,* observed/expected.

**Table E2:** A total of 399 intracardiac cases

Variable	N = 399
Age, mean ± SD	59.1 ± 14.8
STS Score, mean ± SD (n = 317)	1.72 ± 2.18
Intraoperative blood transfusion, n (%)	94 (23.6)
Extubation <6 h, n (%)	279 (69.9)
Hospital LOS (d), mean ± SD	2.43 ± 1.26
ICU LOS (d), mean ± SD	1.28 ± 0.57
Take-back for bleeding, n (%)	18 (4.51)
Conversion, n (%)	7 (1.75)
Postoperative stroke, n (%)	5 (1.25)
Postoperative MI, n (%)	0 (0.00)
Mortality, n (%)	6 (1.50)
Mortality, O/E	0.87

*SD,* Standard deviation; *STS,* Society of Thoracic Surgeons; *LOS,* length of stay; *ICU,* intensive care unit; *MI,* myocardial infarction; *O/E,* observed/expected.

**Table E3:** Eighty epicardial cases

Variable	N = 80
Age, mean ± SD	63.0 ± 13.0
STS Score, mean ± SD	NA
Intraoperative blood transfusion, n (%)	3 (3.75)
Extubation <6 h, n (%)	67 (83.8)
Hospital LOS (d), mean ± SD	2.90 ± 1.58
ICU LOS (d), mean ± SD	1.09 ± 0.57
Take-back for bleeding, n (%)	0 (0.00)
Conversion, n (%)	0 (0.00)
Postoperative stroke, n (%)	1 (1.25)
Postoperative MI, n (%)	1 (1.25)
Mortality, n (%)	1 (1.25)
Mortality, O/E	NA

*SD,* Standard deviation; *STS,* Society of Thoracic Surgeons; *LOS,* length of stay; *ICU,* intensive care unit; *MI,* myocardial infarction; *O/E,* observed/expected; *NA,* not available.

**Table E4:** Thirty-nine other epicardial cases

Variable	N = 39
Age, mean ± SD	49.4 ± 14.5
STS Score, mean ± SD	NA
Intraoperative blood transfusion, n (%)	3 (7.69)
Extubation <6 h, n (%)	37 (94.9)
Hospital LOS (d), mean ± SD	2.72 ± 1.98
ICU LOS (d), mean ± SD	1.15 ± 0.98
Take-back for bleeding, n (%)	0 (0.00)
Conversion, n (%)	0 (0.00)
Postoperative stroke, n (%)	0 (0.00)
Postoperative MI, n (%)	0 (0.00)
Mortality, n (%)	0 (0.00)
Mortality, O/E	0 (0.00)

*SD,* Standard deviation; *STS*, Society of Thoracic Surgeons; *NA,* not available; *LOS,* length of stay; *ICU,* intensive care unit; *MI,* myocardial Infarction; *O/E,* observed/expected.

## Supplementary Data

Video 1 Example of a robotic epicardial procedure: totally endoscopic coronary bypass left internal thoracic surgery/left anterior descending. Video available at: https://www.jtcvs.org/article/S2666-2507(22)00074-8/fulltext.

Video 2 Example of a robotic intracardiac procedure: septal myomectomy for hypertrophic obstructive cardiomyopathy. Video available at: https://www.jtcvs.org/article/S2666-2507(22)00074-8/fulltext.
