# Supplementary figures and images for: Multi-spectrum robotic cardiac surgery: Early outcomes
Source: JTCVS Tech. 2022 Feb 19;13:74–82. doi: 10.1016/j.xjtc.2021.12.018 (PMC9195635; doi:10.1016/j.xjtc.2021.12.018)

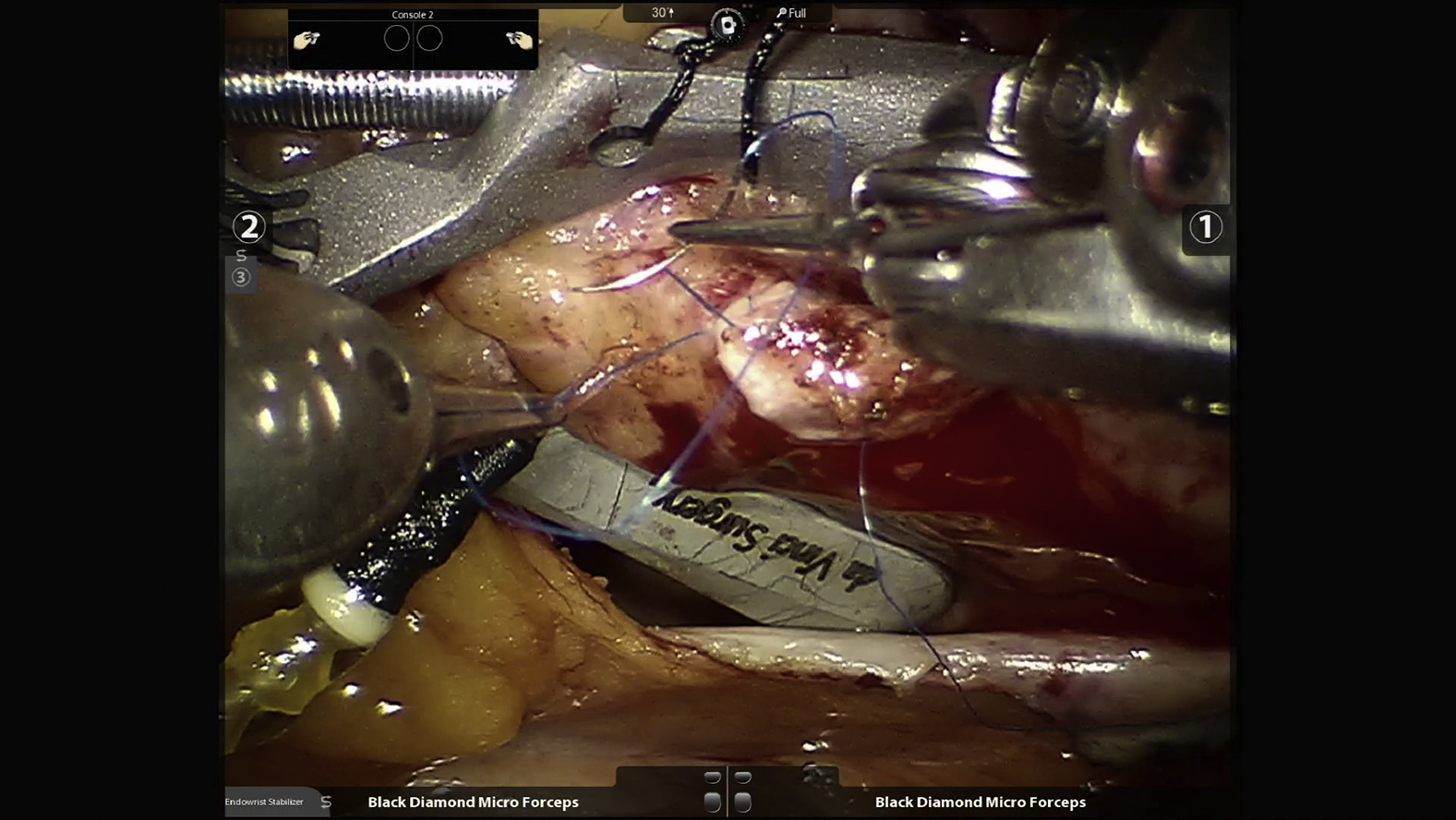

Supplement: Video 1 — Example of a robotic epicardial procedure: totally endoscopic coronary bypass left internal thoracic surgery/left anterior descending. Video available at: https://www.jtcvs.org/article/S2666-2507(22)00074-8/fulltext. [file fx3.jpg]

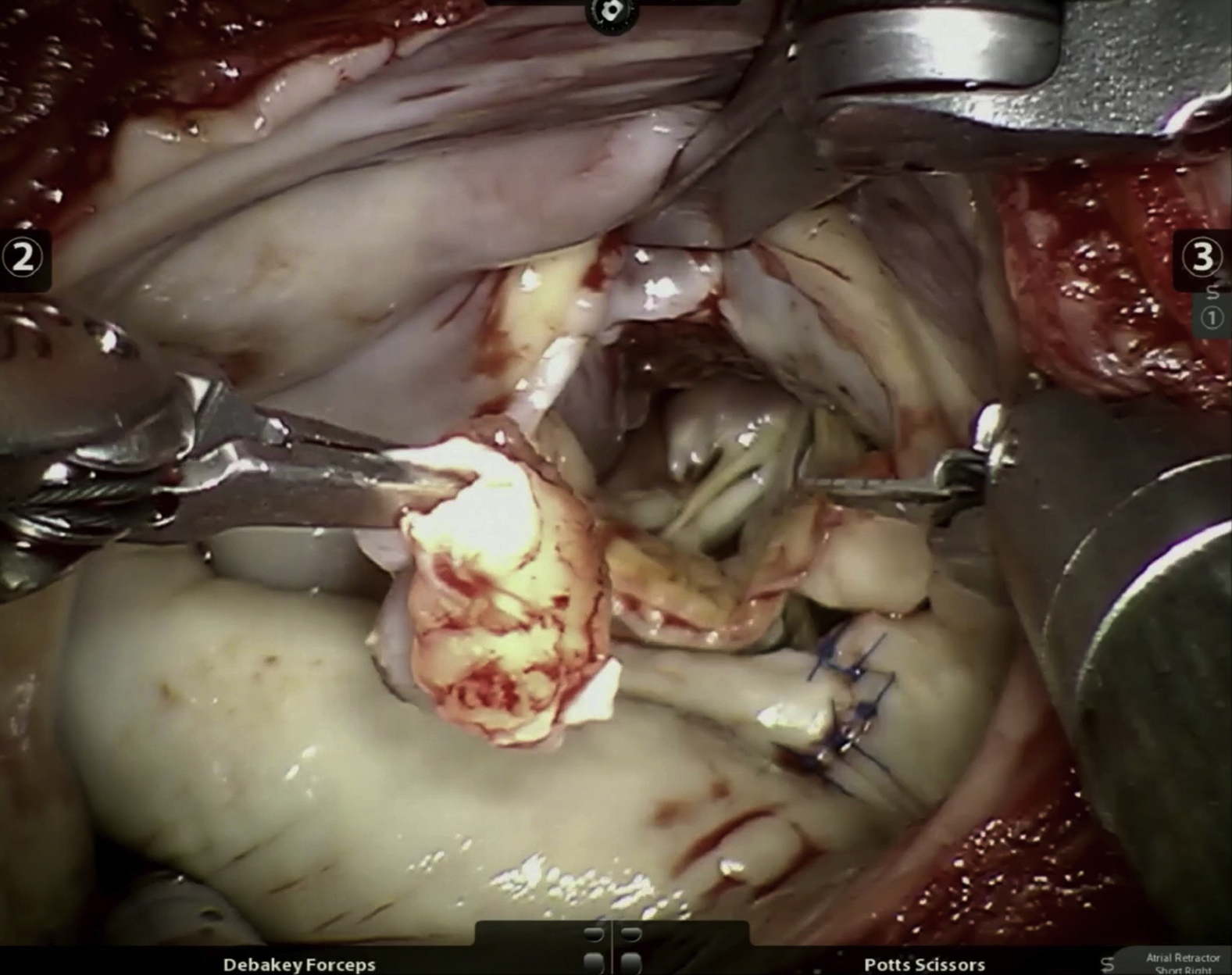

Supplement: Video 2 — Example of a robotic intracardiac procedure: septal myomectomy for hypertrophic obstructive cardiomyopathy. Video available at: https://www.jtcvs.org/article/S2666-2507(22)00074-8/fulltext. [file fx4.jpg]
